# A New Avenue for Classification and Prediction of Olive Cultivars Using Supervised and Unsupervised Algorithms

**DOI:** 10.1371/journal.pone.0044164

**Published:** 2012-09-05

**Authors:** Amir H. Beiki, Saba Saboor, Mansour Ebrahimi

**Affiliations:** 1 Department of Biology, School of Basic Sciences and Bioinformatics Research Group, University of Qom, Qom, Iran; 2 Department of Agricultural Biotechnology, Faculty of Engineering and Technology, IKIU, Qazvin, Iran; Université de Nantes, France

## Abstract

Various methods have been used to identify cultivares of olive trees; herein we used different bioinformatics algorithms to propose new tools to classify 10 cultivares of olive based on RAPD and ISSR genetic markers datasets generated from PCR reactions. Five RAPD markers (OPA0a21, OPD16a, OP01a1, OPD16a1 and OPA0a8) and five ISSR markers (UBC841a4, UBC868a7, UBC841a14, U12BC807a and UBC810a13) selected as the most important markers by all attribute weighting models. K-Medoids unsupervised clustering run on SVM dataset was fully able to cluster each olive cultivar to the right classes. All trees (176) induced by decision tree models generated meaningful trees and UBC841a4 attribute clearly distinguished between foreign and domestic olive cultivars with 100% accuracy. Predictive machine learning algorithms (SVM and Naïve Bayes) were also able to predict the right class of olive cultivares with 100% accuracy. For the first time, our results showed data mining techniques can be effectively used to distinguish between plant cultivares and proposed machine learning based systems in this study can predict new olive cultivars with the best possible accuracy.

## Introduction

Olive (*Olea europaea* L.) has been domesticated by 5800 B.P. [1] probably both in Eastern and Western of the Mediterranean basin [2]–[4]. Archaeological findings revealed that olive cultivation in Iran dates back to more than 2000 years ago [5]. Until recent years, cultivar identification has been based on morphological and agronomic traits. However, the recognition of olive cultivars based on phenotypic characters is often problematic, especially at the early stages of tree development [6]. This has led to great confusion and uncertainty about the current status of olive germplasm in many countries. The ability to discriminate and predict olive cultivars is important for successful breeding programs and improved management of genetic resources [7]. With the development of PCR-based DNA markers such as RAPD [8] SSR [9], AFLPs [10] and SNP [11], marker technology today offers powerful tools to analysis the plant genome. They have enabled the identification of genes and genome associated with the expression of qualitative and quantitative traits and has led to a better understanding of the complex genome of various plants. The use of molecular markers to manage olive germplasm is particularly advantageous, due to the fact that the olive has an exceptionally long juvenile period [12]. Recently, bioinformatics and data mining application have been widely used in interpreting information from biological data. [13]–[16].

The main goal of this work was to construct a molecular database based on RAPD and ISSR markers for olive cultivares and to find specific molecular markers to quickly distinguish between Iranian and foreign olive tree cultivars.

## Materials and Methods

Genomic DNA of five Iranian and five foreign olive (*Olea europaea* L.) cultivars were isolated from freshly harvested young leaves of five plants from IKIU fields of Qazvin University (with the permission from the head; school of agriculture, Qazvin University, Iran; the cultivars have not been designed as protected or endangered species) of each cultivar by Mini prep method. To eliminate the effects of impurity, just these ten cultivares; whom were officially proven by administrative bodies to be pure and the most reliable; were chosen for lab experiments. A total of 14 primers ((AG)_8_T, (AG)_8_C, (GA)_8_T, (GA)_8_C, (GA)_8_A, (CA)_8_G, (AG)_8_CT, (AG)_8_CC, (AG)_8_CA, (GA)_8_CC, (GA)_8_CCY, (AC)_8_YA, (GA)_8_A and (GGAGA)_3_) for inter-simple sequence repeat-polymerase chain reaction (ISSR-PCR) and 14 primers (5′-GTGATCGCAG-3′, 5′-CAATCGCCGT-3′, 5′-GTTTCGCTCC-3′, 5′-AAGACCCCTC-3′, 5′-GGTGACTGTG-3′, 5′-TCTGTGCCAC-3′, 5′-TCGGCGGTTC-3′, 5′-CCGAATTCCC-3′, 5′-CACAGAGGGA-3′, 5′-GTGACGTAGG-3′, 5′-TGAGCGGACA-3′, 5′-CATCCGTGCT-3′, 5′-CCTGGGCTTC-3′, and 5′-GTCCCGTTCA-3′) for random amplified polymorphic DNA were used in the study (Table 1).

**Table 1:** Names and the sequences of ISSR and RAPD marker.

ISSR Primer	Sequence 5^′^–3^′^	Primer ISSR	Sequence 5^′^–3^′^	Primer RAPD	Sequence 5^′^–3^′^	Primer RAPD	Sequence 5^′^–3^′^
**UBC-807**	(AG)_8_T	UBC-835	(AG)_8_CC	OPA-10	GTGATCGCAG	OPA08	GTGACGTAGG
**UBC-808**	(AG)_8_C	UBC-836	(AG)_8_CA	OPA-11	CAATCGCCGT	OPD05	TGAGCGGACA
**UBC-810**	(GA)_8_T	UBC-841	(GA)_8_CC	OPB-01	GTTTCGCTCC	OPD15	CATCCGTGCT
**UBC-811**	(GA)_8_C	UBC-841Y	(GA)_8_CCY	OPE-06	AAGACCCCTC	OPDP6	TCGGCGGTTC
**UBC-812**	(GA)_8_A	UBC-856	(AC)_8_YA	OPE-16	GGTGACTGTG	OPD01	CCTGGGCTTC
**UBC-818**	(CA)_8_G	UBC-868	(GA)_8_A	OPF-05	CCGAATTCCC	OPA01	TCTGTGCCAC
**UBC-834**	(AG)_8_CT	UBC-880	(GGAGA)_3_	OPA-04	CACAGAGGGA	OPA00	GTCCCGTTCA

ISSR-PCR was conducted in a reaction volume of 15 µl containing 30 ng template DNA, 0.2 µmol/L primer, 200 µmol/L each dNTP, 10 mmol/L Tris-Cl (pH 8.3), 50 mmol/L KCl, 2.0 mmol/L MgCl2, and 1 U of Taq polymerase. PCR amplification conditions were set as initial denaturation at 94°C for 5 min, 40 cycles of denaturation at 94°C for 1 min, annealing at 50°C for 1 min, extension at 72°C for 2 min, and a final extension at 72°C for 7 min. PCR was performed in 96-well plate thermal cycler (Eppendorf, Germany). The amplified products were mixed with loading dye (0.4 g/ml sucrose and 2.5 mg/ml bromophenol blue), resolved on 18 mg/ml.

**Table 2:** The numbers and the averages of most important alleles (fragments) selected by different attribute weighting algorithms.

Alleles (fragments)	Number of attribute weightings	Average of attribute weightings	Alleles (fragments)	Number of attribute weightings	Average of attribute weightings
**UBC841a4**	10	0.982	**UBC841Ya8**	10	0.737
**UBC868a7**	10	0.982	**OPD16a1**	10	0.737
**UBC841a14**	10	0.680	**UBC807a13**	10	0.735
**OPA0a21**	10	0.680	**OPA0a8**	10	0.735
**OPD16a**	10	0.680	**OPD15a1**	10	0.735
**OP01a1**	10	0.720	**OPD15a2**	10	0.735
**BC807a12**	10	0.712	**UBC810a12**	10	0.688
**UBC810a13**	10	0.712	**UBC868a8**	10	0.688

The RAPD technique consists of preferential amplification of random sequences by PCR. In this assay, 10 different primers were used (Table 1). Each 25 µL PCR reaction mixture consisted of 50 ng genomic DNA, 0.2 mMdNTPs, 2 mM MgCl2, 10pmol primer, 2.5 µL 10× Taq buffer, and 1 unit of Taq polymerase. Samples were subjected to the following thermal profile: 4 min of denaturing at 94°C, forty-five cycles of three steps: 30 s of denaturing at 94°C, 1 min of annealing at 36°C, and 2 min of elongation at 72°C, with a final elongation step of 7 min 72°C. Separation of the amplified fragments was performed on 1.2% (w/v) agarose gels, TAE 1x] at 80V during 2 h. The gels were stained with ethidium bromide for visualizing the RAPD and ISSR fragments. The fragments between 200 and 4k base pair (bp) were visually scored as present (1) or absent (0).

A dataset of 10 cultivar with 402 RAPD and ISSR reproducible fragments or attributes prepared and was imported into RapidMiner software [RapidMiner 5.2, Rapid-I GmbH, Stochumer Str. 475, 44227 Dortmund, Germany]. Then, the steps detailed below were applied to this dataset.

### Data Cleaning

Useless attributes were removed from the dataset. Nominal attributes were regarded as useless when the most frequent values were above or below per cent of all examples. After cleaning, this database was labelled the final cleaned database (FCdb).

### Attribute Weighting

To identify the most important features that contribute to different olive cultivars, 10 different algorithms of attribute weightings (*Information gain, Information Gain ratio, Rule, Deviation, Chi squared statistic, Gini index, Uncertainty, Relief, SVM and PCA* ***)*** were used (for more information see [14], [15], [17]).

**Table 3:** The attribute weighting models and the numbers of important protein features selected by each model and the most important variables selected by each attribute weighting algorithms.

Attribute Weighting	Number of Variable	Important variable
*Information gain*	16	UBC841A4; UBC868A7; OPA0A21; OPA0A8
*Information gain Ratio*	16	UBC841A4; UBC868A7; OPA0A21; OPA0A8
*Rule*	57	UBC841A4; UBC868A7; OPA0A21; OPA0A8
*Deviation*	160	UBC808A13; UBC808A15; OPA10A10; OPA11A7
*Chi squared*	2	UBC841A4; UBC868A7; UBC841A14; OPA0A21
*Gini index*	16	UBC841A4; UBC868A7; UBC841A14; OPA0A21
*Uncertainty*	16	UBC841A4; UBC868A7; UBC841A14; OPA0A21
*Relief*	16	UBC841A4; UBC868A7; UBC841A14; OPA0A21;
*SVM*	115	OPD1A1; OPA0A7; UBC841A4; UBC868A7
*PCA*	76	UBC834A7; UBC834A8; UBC856A3; UBC856A6;
**FCdb**	400	

### Attribute selection

Application of attribute weighting models on the dataset gave each alleles attribute (feature) a value between 0 and 1, which revealed the importance of that attribute with regards to a target attribute (Iranian or foreign cultivar). All variables with weights higher than 0.50 were selected and 10 new datasets created. These newly formed datasets were named according to their attribute weighting models (*Information gain, Information gain ratio, Rule, Deviation, Chi Squared, Gini index, Uncertainty, Relief, SVM and PCA*) and were subjected to subsequent supervised or unsupervised models. Each supervised or unsupervised model was performed 11 times; the first time it ran on the main dataset (FCdb) and then on the 10 newly formed datasets from attribute weighting and selection.

### Unsupervised Clustering Algorithms

The clustering algorithms listed below were applied on the 10 newly created datasets (generated as the outcomes of 10 different attribute weighing algorithms) as well as the main dataset (FCdb).

#### K-Means

This operator uses kernels to estimate the distance between objects and clusters. Because of the nature of kernels, it is necessary to sum over all elements of a cluster to calculate one distance.

#### K-Medoids

This operator represents an implementation of k-Medoids. This operator will create a cluster attribute if it is not yet present.

#### Support vector clustering (SVC)

This operator represents an implementation of Support Vector algorithm. This operator will create a cluster attribute if not present yet.

#### Expectation maximization (EM)

This operator represents an implementation of the EM-algorithm.

**Figure 1:**
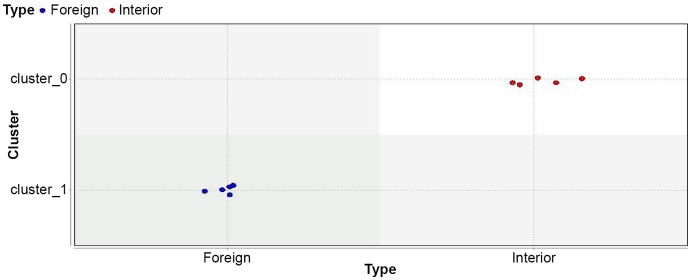
Application of K-Medoids to the SVM was able to categorize each cultivar into right cluster.

**Table 4:** The numbers of olive cultivars correctly predicted by three different unsupervised clustering algorithms ran on all databases.

Database		K-Means		K-Medoids		SV	
	Cultivar	Predicted Number	Correct predicted Number	Predicted Number	Correct predicted Number	Predicted Number	Correct predicted Number
**FCdb**	Iranian	10	5	10	5	0	0
	Foreign	0	0	0	0	10	5
**Chi Square**	Iranian	10	5	10	5	0	0
	Foreign	0	0	0	0	10	5
**Deviation**	Iranian	7	4	5	1	Noise	–
	Foreign	3	2	5	1	Noise	–
**Gini Index**	Iranian	10	5	10	5	0	0
	Foreign	0	0	0	0	10	5
**Info Gain**	Iranian	10	5	10	5	0	0
	Foreign	0	0	0	0	10	5
**Info Gain Ratio**	Iranian	10	5	10	5	0	0
	Foreign	0	0	0	0	10	5
**PCA**	Iranian	7	4	5	4	Noise	–
	Foreign	3	2	5	4	Noise	–
**Relief**	Iranian	10	5	10	5	0	0
	Foreign	0	0	0	0	10	5
**Rule**	Iranian	10	5	10	5	0	0
	Foreign	0	0	0	0	10	5
**SVM**	Iranian	7	5	5	5	Noise	–
	Foreign	3	0	5	5	Noise	–
**Uncertainty**	Iranian	10	5	10	5	0	0
	Foreign	0	0	0	0	10	5

### Supervised Classification

Three classes of supervised classification (*Decision Trees*, SVM and *Baysian* models) applied as follows. To calculate the accuracy of each model, 10-fold cross validation [18] is used to train and test models on all patterns. To perform cross validation, all the records were randomly divided into five parts; four sets were used for training and the 5th one for testing. The process was repeated five times and the accuracy for true, false and total accuracy calculated. The final accuracy is the average of the accuracy in all five tests.

**Figure 2:**
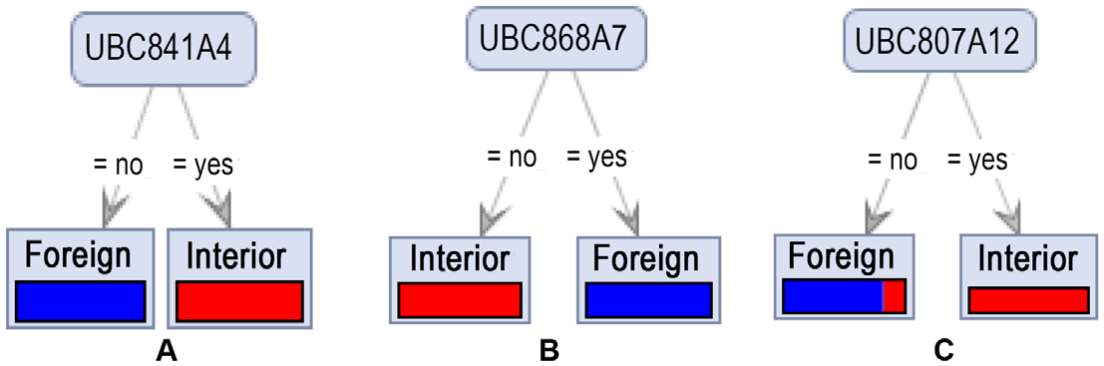
Decision Tree generated from three models ran with *Gini Index* criterion. As may be inferred from the figure, UBC841A4 and UBC868A7 fragments were the most important attribute alleles in distinguishing Iranian from foreign cultivars.

### Decision Trees

Six tree induction models including *Decision Tree, Decision Tree Parallel, Decision Stump, Random Tree, ID3 Numerical and Random Forest* were run on the main dataset (FCdb). Each tree induction model ran with the following four different criteria: *Gain Ratio, Information Gain, Gini Index and Accuracy*. In addition, a *weight-based parallel decision tree* model, which learns a pruned decision tree based on an arbitrary feature relevance test (attribute weighting scheme as inner operator), was run with 13 different weighing criteria (*SVM, Gini Index, Uncertainty, PCA, Chi Squared, Rule, Relief, Information Gain, Information Gain Ratio, Deviation, Correlation, Value Average, and Tree Importance*). The accuracy of each tree computed based on the previous explanation.

**Table 5:** The accuracies, precisions and recalls of tree induction models on Final Cleaned database (FCdb) computed on 5-fold cross validation.

Models	Algorithm	Gain Ratio	Information Gain	Gini Index	Accuracy
**Decision Tree**	**Overall Accuracy**	70	70	70	70
	**Iranian Recall**	60	60	60	60
	**Foreign Recall**	80	80	80	80
	**Iranian Precision**	75	75	75	75
	**Foreign Precision**	66.7	66.7	66.7	66.7
**Decision Tree Parallel**	**Overall Precision**	70	70	50	50
	**Iranian Recall**	60	60	0	0
	**Foreign Recall**	80	80	100	100
	**Iranian Precision**	75	75	unknown	unknown
	**Foreign Precision**	66.7	66.7	50	50
**Decision Stump**	**Overall Precision**	70	70	50	50
	**Iranian Recall**	60	60	0	0
	**Foreign Recall**	80	80	100	100
	**Iranian Precision**	75	75	unknown	Unknown
	**Foreign Precision**	66.7	66.7	50	50
**Random Forest**	**Overall Precision**	70	70	70	70
	**Iranian Recall**	60	60	60	60
	**Foreign Recall**	80	80	80	80
	**Iranian Precision**	75	75	75	75
	**Foreign Precision**	66.7	66.7	66.7	66.7
**Random Tree**	**Overall Precision**	70	70	70	70
	**Iranian Recall**	60	60	60	60
	**Foreign Recall**	80	80	80	80
	**Iranian Precision**	75	75	75	75
	**Foreign Precision**	66.7	66.7	66.7	66.7

### Support Vector Machine Approach

Support Vector Machines (SVMs) are popular and powerful techniques for supervised data classification and prediction; so SVM, LibSVM, SVM Linear and SVME used here to implement different models to predict olive cultivars based on Iranian - foreign features. Briefly, main database (FCdb) transformed to SVM format and scaled by grid search (to avoid attributes in greater numeric ranges dominating those in smaller numeric ranges) and to find the optimal values for operator parameters. To prevent overfitting problems, 5-fold cross validation applied. Dataset divided into 5 parts and 4 parts used as training set and the last part as testing set, the procedure repeated for 10 different testing sets and the average of accuracy computed. RBF kernel which nonlinearly maps samples into a higher dimensional space and can handle the case when the relation between class labels and attributes is nonlinear used to run the model. Other kernels such as linear, poly, sigmoid and pre-computed were also applied to the dataset to find the best accuracy.

### Naïve Bayes

Naïve Bayes based on Bayes conditional probability rule is used for performing classification tasks. When the sample sizes tend to be small (as in our experiments with just 5 cultivars in each class), a Bayesian approach can be applied for classification problems with far more predictors than samples; the same have been widely used before (for more details see [19], [20]. Naïve Bayes assumes the predictors are statistically independent which makes it an effective classification tool that is easy to interpret. Two models, Naïve base (returns classification model using estimated normal distributions) and Naïve base kernel (returns classification model using estimated kernel densities) used and the model accuracy in predicting the right Iranian - foreign computed as stated before.

**Figure 3:**
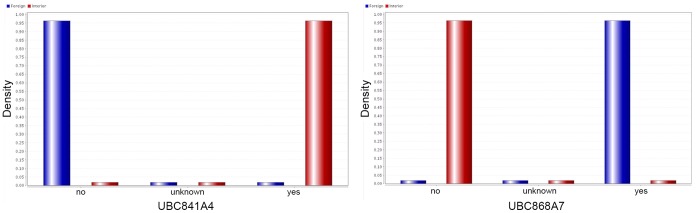
Kernel distribution model distinguishing between two classes of Olive cultivares based on allele attribute type.

## Results

As mentioned in Materials and Methods, the initial dataset contained 10 cultivars with 400 RAPD and ISSR reproducible fragments (attributes). Following removal of duplicates, useless attributes, and correlated features (data cleaning) 312 features remained; meaning these attribute fragments were polymorphic, ranging in size from 100 to 3000 bp.

### Attribute Weighting

The number of attributes gained weights higher than 0.5 in each weighting model were as follows: *PCA* 76, *SVM* 114, *Relief* 16, *Uncertainty* 16, *Gini index* 16, *Chi Squared* 16, *Deviation* 244, *Rule* 57, *Gain ratio* 16 and *Info gain ratio* 16 (Table 2). The details of the most important attributes have been presented in Table 3.

### Unsupervised Clustering Algorithms

Three different unsupervised clustering algorithms (K-Means, K-Medoids and SVC) were applied on ten datasets created using attribute selection (weighting) algorithms. Some models, such as the application of the SVC algorithm on ten datasets were unable to differentiate interior from foreign cultivars (Table 4). Application of the K-Means and K-Medoids on all databases (except D*eviation*, *PCA* and *SVM* databases) was unable to assign any cultivars into its correct class. K-Means and K-Medoids methods correctly predicted Iranian and foreign cultivares into the right cluster, respectively. So the combination of K-Means and K-Medoids with *Deviation*, *PCA* and *SVM* databases can effectively cluster the right cultivars. Interestingly, just application of K-Medoids method to the *SVM* dataset was able to categorize cultivars into the correct cluster (Figure 1).

### Supervised Classification

#### Decision trees

All 176 tree induction tree (4 models: *Decision Stump, Decision Tree, Decision Parallel* and *Random Forest Tree* each with 4 different criteria - *Gain ratio, Information gain, Gini index and Accuracy* – run on 11 different datasets) were able to produce the same trees (Figure 2A). The accuracies and precisions of decision tree algorithms were nearly the same (Table 4). UBC841A4 allele was the most important attribute used to build the trees. When this attribute has removed from datasets, interestingly again a simple decision tree were generated by all models (Figure2 B). So, if the fragment of UBC841A4 presents, the cultivar is foreign origin, otherwise, if the fragment of UBC868A7 detects, the cultivar origin is from Iran. When these two attributes were removed from databases, another simple decision tree generated (Figure2 C). The figure shows that UBC807A12 fragment can predict Iranian cultivars with little accuracy.

As shown in Table 5, the overall accuracies for tree induction models were generally high enough for all algorithms ran with various criteria which are a very sharp increase in model accuracy and performance. Almost in all models and algorithms, precision of Iranian cultivar prediction were better than foreign cultivar prediction except when *Decision Stump Tree* and *Decision Tree Parallel* models ran with *Accuracy* and *Gini Index*. In these cases induced trees were not able to predict Iranian cultivars.

#### SVM approach

The total accuracy predicted by different SVM methods (when Gamma and C were 0.0065 and 10, respectively) reached 100%.The overall accuracies of different *SVM* models ran with different database were in the range of 0–100%, while the same accuracies for *SVM* and *SVMLinear* model ran on all databases were over 80%.

#### Naïve bayes

The accuracies of *Naïve base* and *Naïve Bayes Kernel* models ran on all databases were at maximum point (100%) except when applied on *FCdb*, *PCA* and *Deviation* databases which fell down to 80±0.43%. *Kernel Distribution* model for label attribute (foreign and Iranian) on the base of selected features has shown in figure 3. As shown in figure 3, two fragment attributes can simply predict Iranian from foreign cultivars.

## Discussion

Accurate and rapid identification of clones, varieties, or species is especially important in vegetatively propagated plants. The official key for identification of olive varieties is based on morphological criteria [21], [22] although they are influenced by environmental conditions. However, molecular markers are environment-independent and efficient to identify olive cultivares and to detect synonymous and homonymous [23]–[25]. With the light of recent molecular genetic studies, another aspect of olive identification has become “rich genetic diversity” [26], [27]. This genetic diversity at cultivar level is important due to significant economic aspects such as yield and chemical and/or aromatic composition of fruit and olive oil [27]–[30]. To resolve the genetic complexity and to differentiate cultivars from one another different molecular systematic studies have been conducted [31], [32]. Herein, we aimed to determine the most important features contribute to the clustering, classification and prediction of Iranian from foreign cultivars based on genetic alleles. Various modelling techniques were applied to study more than 311 attribute alleles of this family.

Knowledge discovery through pattern finding in data is central to modern molecular biology, with thousands of databases and similar numbers of tools for data processing. Any data analysis in molecular biology involves gathering and processing data from many sources, even before the analysis for the central biological question takes place. The goal of the clustering algorithms (unsupervised pattern ) is to figure out the underlying similarities among a set of feature vectors, and to cluster similar vectors together [14], [15], [33], while decision trees are very popular tools for classification [34]. The attractiveness of decision trees is due to the fact that, decision trees represent rules. Rules can readily be expressed so that humans can understand them. Decision trees provide the information about which attributes are most important for prediction or classification [15], [16], [35], [36].

When the number of variables or attributes is sufficiently large, the ability to process units is significantly reduced. Data cleaning algorithms were used to remove correlated, useless or duplicated attributes which results in a smaller database [14]–[16]. More than 20% of the attribute alleles discarded when these algorithms were applied on the original dataset. Each attribute weighting system uses a specific pattern to define the most important features by feature selection [37]–[39]. Thus, the results may be different [40], as has been highlighted in previous studies [13]–[17].

UBC841A4, UBC868A7 and UBC841YA8 fragments from ISSR markers and OPE16A1, OPA0A8 and OPD15A1 fragments from RAPD markers were the most important feature to distinguish Iranian from foreign cultivars, as defined by the entire attribute weighting algorithms (Table 1). Several previous studies have used these markers for fingerprinting identification and characterization of genomic region in olives [41]–[50] but to our knowledge, this is the first study reports the use of supervised and unsupervised methods and predictive models to identify the Iranian from foreign olive cultivars with a precision rate up to 100%.

Unsupervised clustering algorithms have been widely used in a various areas in the biological sciences, including proteomics, predicting gene function and genomics [14], [15], [34], [51], metabolomics [52], [53] and transcriptomics [51]. These methods are preferred for prediction because they are capable of discovering structure by exploring similarities and differences between individual data points in a given data set. Here, we used four different unsupervised clustering methods (*K-Means, K-Medoids, SVC and MEMC*) on 11 datasets created from RAPAD and ISSR allele attributes, which were assigned high weights. The performances of these algorithms varied significantly, usually these algorithms work well when the numbers of classes to be clustered are small (less than 4). Here we have only two classes, foreign and Iranian cultivars and it is expected that these algorithms are suitable for this condition and there is no need more complex clustering. The results showed that the performance of k-Medoids by SVM algorithm was better than the others. It is able to classify Iranian and foreign cultivar into the correct classes. Cluster analysis techniques are concerned with exploring data sets to assess whether or not they can be summarized meaningfully in terms of a relatively small number of groups or clusters of objects or individuals which resemble each other and which are different in some respects from individuals in other clusters. Standard clustering methods have been developed in many directions to encompass realistic situations. Application fields such as genetics, combined with increasing computing power, have prompted some of these developments [14], [34], [36], [54]. The classification of plants has clearly played an important role in the fields of biology [31], [55], [56].

All prediction trees generated by tree induction models had simple shape with two branches. The ability of various decision tree induction models applied in this study to correctly and effectively classify cultivars based on fragment attributes were identical. Therefore all tree induction algorithms may be effectively used as suitable tools to classify those olive cultivars with maximum accuracies. As shown in Table 5, the overall accuracies for tree induction models were generally high enough for all algorithms. Precision of Iranian cultivar prediction is more than foreign cultivar prediction except when *Decision Tree Stump* and *Decision Tree Parallel* ran with *Accuracy* and *Gini Index*. In these cases trees did not predict Iranian cultivars.

The support vector machine is a learning machine for two-group classification problems and have been widely employed by researchers in different areas of science, including genomics, proteomics, metabonomics**,** researches [15]–[17], [34]–[36]. According to this study, SVM has shown promising capability for prediction of Iranian and foreign olive cultivars. Therefore, SVM is expected to be a potential eligible algorithm which can be employed for classification and prediction of any two classes of olive cultivar.

### Conclusion

The past decade has been witness to a tremendous growth in bioinformatics, as a combination of molecular biology, computer science, mathematics and statistics. Such growth has been accelerated by the ever-expanding genomic and proteomic databases, which are themselves the result of rapid technological advances in molecular genetics. Statistics and bioinformatics have, so far, played important roles in this scientific revolution. Molecular genetics techniques have made it clear that major events in the life of a cell are regulated by factors that alter the expression of the gene. Huge amounts of data accumulated in this field need new tools other than classical statistical methods to interpret and manipulate them; bioinformatics tools have served great job in this field. Herein, various supervised and unsupervised tools applied to identify groups of alleles with similar patterns of expression to find suitable tools to correctly cluster 10 olive cultivars. Up to our knowledge, this is the first report showing the importance and application of bioinformatics algorithms in classifying olive cultivares and the first designed machine learning and predictive system to predict the cultivares with the maximum possible accuracy.
